# C-Jun recruits the NSL complex to regulate its target gene expression by modulating H4K16 acetylation and promoting the release of the repressive NuRD complex

**DOI:** 10.18632/oncotarget.3988

**Published:** 2015-05-04

**Authors:** Yan Liu, Yuehong Long, Zhaobin Xing, Daoyong Zhang

**Affiliations:** ^1^ College of Life Sciences, Hebei United University, Tangshan, China; ^2^ Department of Biochemistry and Molecular Biology, The Pennsylvania State University, University Park, PA, USA

**Keywords:** C-Jun, NSL complex, H4K16 acetylation, NuRD complex, phosphorylation

## Abstract

The proto-oncogene c-Jun plays essential roles in various cellular processes, including cell proliferation, cell differentiation, and cellular apoptosis. Enormous efforts have been made to understand the mechanisms regulating c-Jun activation. The males absent on the first (MOF)-containing non-specific lethal (NSL) complex has been shown to positively regulate gene expression. However, the biological function of the NSL complex is largely unknown. Here we present evidence showing that c-Jun recruits the NSL complex to c-Jun target genes upon activation. The NSL complex catalyzes H4K16 acetylation at c-Jun target genes, thereby promoting c-Jun target gene transcription. More interestingly, we also found that the NSL complex promotes the release of the repressive NuRD complex from c-Jun target genes, thus activating c-Jun. Our findings not only reveal a new mechanism regulating c-Jun activation, but also identify the NSL complex as a c-Jun co-activator in c-Jun-regulated gene expression, expanding our knowledge of the function of the NSL complex in gene expression regulation.

## INTRODUCTION

C-Jun, the major AP-1 transcription factor family member, is activated by a broad range of extracellular signals, including growth factors, cytokines and extracellular stresses [1]. C-Jun plays essential roles in a variety of cellular processes, ranging from cell proliferation and differentiation to tumorigenesis and cellular apoptosis [2-5]. The overexpression of c-Jun has been observed in many types of carcinomas, including nasopharyngeal carcinomas (NPC), solid squamous cell carcinomas (SCC), glioblastoma, and other cancer types [6-13]. C-Jun has also been shown to be essential in the development of skin and liver tumors [14, 15]. Several studies have demonstrated that c-Jun possesses the potential to induce malignant transformation of several cell lines [16, 17]. C-Jun exerts its regulatory role in cellular proliferation and transformation by regulating proliferation-stimulating genes, such as *EGFR, KGF, CyclinD1,* and *CDC2* [18-21]. In addition, c-Jun also downregulates the tumor suppressor gene *p53* to inhibit apoptosis [22, 23]. Moreover, c-Jun enhances angiogenesis and invasiveness by regulating *Proliferin* and *CD44* respectively [24, 25].

The activation of c-Jun requires the phosphorylation of residue Ser63 and Ser73 in its transactivation domain by the c-Jun N-terminal kinase (JNK) [26-28]. The phosphorylation of c-Jun by JNK activates c-Jun through a combinatorial mechanism. First, the phosphorylation of c-Jun has been shown to potentiate its transcriptional activity [26, 27, 29]. In addition, phosphorylation also regulates the nuclear localization of c-Jun and its cofactors, which may be essential for c-Jun activation [30, 31]. c-Jun protein is unstable in vivo and the poly-ubiquitination of c-Jun leads to its degradation. The phosphorylation of c-Jun has been shown to increase its half-life by reducing its poly-ubiquitination levels, thus prolonging its effect on gene expression in response to extracellular stimuli [32, 33]. In addition, the phosphorylation of c-Jun also regulates its interaction with other transcription regulators. For instance, the phosphorylation of c-Jun by JNK has been reported to cause its dissociation from a repressive complex that contains histone deacetylase 3 (HDAC3), thus increasing its transcriptional activity [34]. More recently, a study showed that the transcriptional activity of c-Jun is repressed by a histone deacetylase-containing repressor complex, the Nucleosome Remodeling Deacetylase (NuRD) and the phosphorylation of c-Jun by JNK led to its dissociation from the NuRD complex, thus leading to the activation of c-Jun [35].

In this study, we demonstrate that phosphorylated c-Jun interacts with the non-specific lethal (NSL) histone acetyltransferase (HAT) complex, a males absent on the first (MOF)-containing chromatin-modifying complex. The recruitment of the NSL complex to c-Jun target gene not only catalyzes H4K16 acetylation to promote gene transcription, but also promotes the release of the NuRD complex from c-Jun target gene, leading to the activation of c-Jun target genes.

## RESULTS

### C-Jun activation dramatically increases H4K16ac levels at the promoters of c-Jun target genes

C-Jun has previously been shown to be engaged in corepressor complexes, which repress c-Jun transcriptional activity [35]. Upon activation, c-Jun is released from the repressive complex, leading to its target gene expression [35]. However, little is known about the c-Jun coactivator in c-Jun-activated gene expression. Here, we aimed to identify the c-Jun coactivator. Many histone modifications have been implicated in gene activation. In the present study, we first tried to examine the change in specific histone modifications that correlate with gene activation in the promoters of c-Jun target genes upon c-Jun activation.

**Figure 1:**
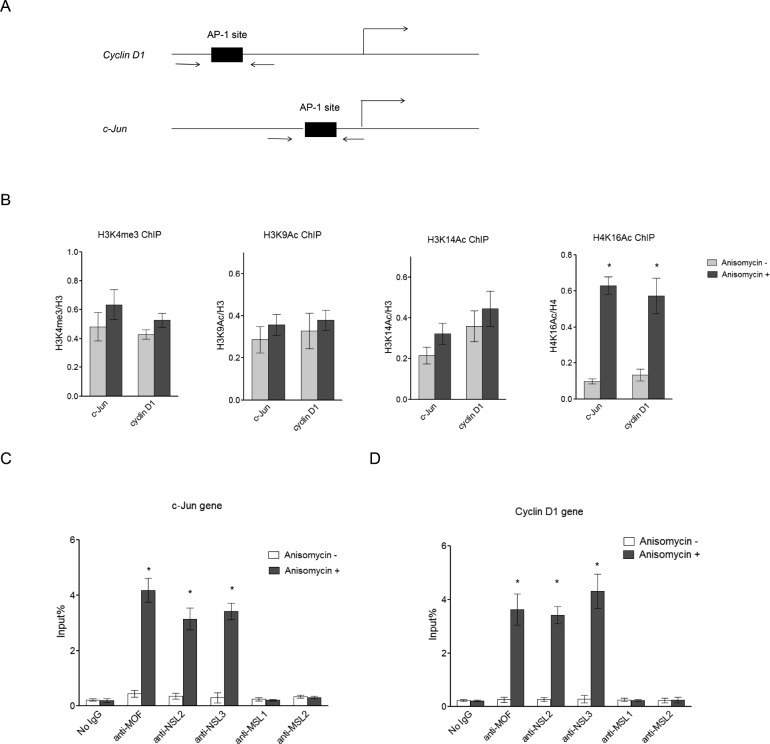
NSL complex binds to c-Jun target genes upon c-Jun activation **A.** Diagram of the *c-Jun* gene locus and *Cyclin D1* gene locus, which are both c-Jun target genes. Arrows indicate the primers used in ChIP assay. **B.** H4K16 acetylation levels at c-Jun target genes are dramatically increased upon c-Jun activation. HEK-293 cells were treated with Anisomycin for 30 minutes to activate c-Jun. The cells were then subjected to ChIP analysis using the indicated antibodies. Untreated cells were used as a control. The data are presented as mean ± standard deviation of 3 independent experiments. *P < 0.001. **C.**, **D.** NSL complex binds to c-Jun target genes upon c-Jun activation. HEK-293 cells were treated with Anisomycin for 30 minutes to activate c-Jun. The cells were then subjected to ChIP analysis using the indicated antibodies. Untreated cells were used as a control. The data are presented as mean ± standard deviation of 3 independent experiments. *P < 0.001.

Several histone modifications have been reported to be enriched in actively transcribed genes, including H3K4me3, H3K9ac, H3K14ac, and H4K16ac [36]. Here, we first measured these histone modifications at the promoter region of the c-Jun target genes, *c-Jun* and *Cyclin D1,* before and after Anisomycin treatment, which induces c-Jun activation (Figure 1A). As seen in Figure 1B, upon c-Jun activation we observed a dramatic increase in H4K16 acetylation levels at the promoters of c-Jun target genes, while other active histone markers were only slightly increased. This result suggests that H4K16ac might play an important role in extracellular signal-induced c-Jun target gene expression, and that the histone acetyltransferase responsible for H4K16 acetylation is involved in c-Jun activated gene expression. MOF has been shown to be the major histone acetyltransferase responsible for H4K16 acetylation in mammals. Therefore, we further examined the presence of MOF at the promoter region of *c-Jun* and *Cyclin D1* after c-Jun activation. The result showed that MOF is recruited to the promoter of *c-Jun* and *Cyclin D1* upon c-Jun activation (Figure 1C and 1D), suggesting that the observed increase in H4K16 acetylation at the promoters of c-Jun target genes is catalyzed by MOF.

There are two distinct MOF-containing complexes in mammals and *Drosophila*, non-specific lethal (NSL) complex and male-specific lethal (MSL) complex [37, 38]. To determine which complex is involved in c-Jun activated gene expression, we measured the occupancy of MSL complex-specific subunits and NSL complex-specific subunits at the promoters of c-Jun target genes upon c-Jun activation. The data above showed that the NSL complex is specifically recruited to the c-Jun target gene promoter upon c-Jun activation (Figure 1C and 1D). This is consistent with previous studies that reported that the NSL complex is primarily localized to the gene promoter while the MSL complex is enriched in the gene body [38]. Collectively, the data above showed that c-Jun activation increased H4K16 acetylation levels and the binding levels of the NSL complex at the promoters of c-Jun target genes, indicating a potential regulatory role of the NSL complex and H4K16 acetylation in c-Jun regulated gene expression.

### C-Jun recruits the NSL complex to the promoters of its target genes upon activation

**Figure 2:**
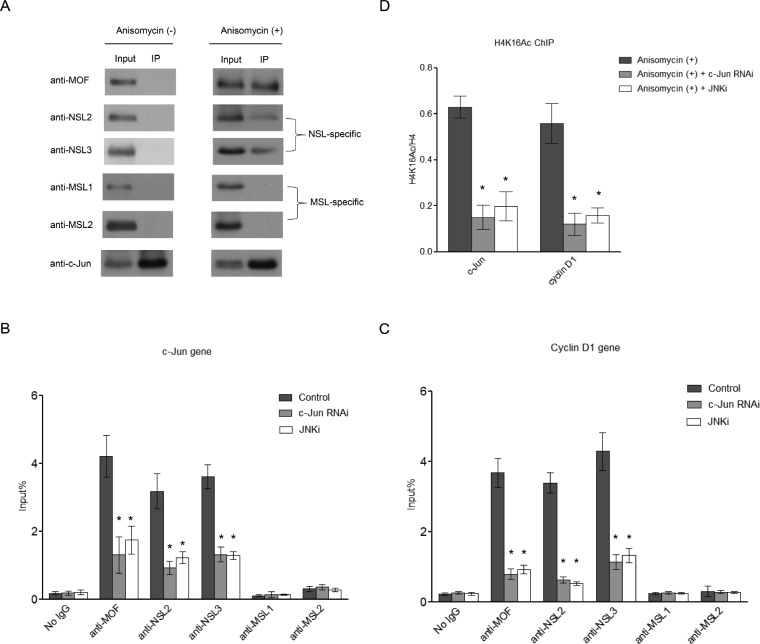
c-Jun recruits the NSL complex to its target genes upon c-Jun activation **A.** Activated c-Jun co-immunoprecipitates with the NSL complex, but not the MSL complex. HEK-293 cells were treated with or without Anisomycin for 30 minutes. Cell lysates obtained from these cells were subjected to immunoprecipitation using anti-c-Jun antibody, followed by western blot assay using the indicated antibodies. **B.**, **C.** The phosphorylation of c-Jun is required for the recruitment of the NSL complex to c-Jun target genes. HEK-293 cells were treated with either siRNAs specific to c-Jun or a JNK inhibitor followed by treatment with Anisomycin. The resultant cells were then subjected to ChIP analysis using antibodies as indicated. The cells treated with only Anisomycin were used as a control. The data are presented as mean ± standard deviation of 3 independent experiments. *P < 0.01. **D.** H4K16 acetylation levels at c-Jun target genes are dramatically reduced upon c-Jun depletion or JNKi treatment. HEK-293 cells were treated with either siRNAs specific to c-Jun or JNK inhibitor, followed by treatment with Anisomycin. The resultant cells were then subjected to ChIP analysis using antibodies as indicated. The cells treated with only Anisomycin were used as a control. The data are presented as mean ± standard deviation of 3 independent experiments. *P < 0.01.

The observation that the NSL complex binds to the promoters of c-Jun target genes prompted us to ask whether the recruitment of the NSL complex is directed by c-Jun. To address this question, we investigated the interaction between c-Jun and the NSL complex. As shown in Figure 2A, after Anisomycin treatment c-Jun co-immunoprecipitated with MOF, along with NSL complex-specific subunits, whereas no MSL complex-specific subunits were detected. This is not due the change in protein levels of the NSL complex components after Anisomycin treatment (Figure S1). These data indicate that c-Jun specifically associates with the NSL complex. Interestingly, c-Jun only co-immunoprecipitates with the NSL complex after Anisomycin treatment, suggesting that the NSL complex interacts with phosphorylated c-Jun, but not unphosphorylated c-Jun.

We further investigated whether the recruitment of the NSL complex to c-Jun target genes is dependent on c-Jun. To answer this question, we first depleted c-Jun in HEK-293 cells by RNA interference (RNAi) (Figure S2). The resultant cells were treated with Anisomycin before being subjected to ChIP analysis to measure the occupancy of the NSL complex at c-Jun target genes. As shown in Figure 2B and 2C, the binding of MOF and NSL complex-specific subunits NSL2 and NSL3 were significantly reduced upon c-Jun depletion. Accordingly, reduced H4K16 acetylation was also observed in c-Jun-depleted cells (Figure 2D). This result indicates that the recruitment of the NSL complex to c-Jun target genes is dependent on c-Jun. To further address whether the recruitment of NSL complex is dependent on c-Jun phosphorylation, we treated HEK-293 cells with a JNK inhibitor (JNKi), which inhibits c-Jun phosphorylation mediated by JNK. We observed that the JNKi treatment disrupted the interaction between c-Jun and the NSL complex, suggesting that the phosphorylation of c-Jun is required for this interaction (Figure S3). The subsequent ChIP analysis showed that JNKi treatment greatly reduced the binding of the NSL complex, as well as H4K16 acetylation at the promoters of c-Jun target genes (Figure 2B, 2C, and 2D). This is also not due to the decreased expression of the NSL complex components upon c-Jun RNAi or JNKi treatment, because no significant change in protein levels of the NSL complex component was observed under the conditions used here (Figure S1). Together, these data demonstrate that c-Jun recruits the NSL complex to the promoter of its target genes and this recruitment is dependent on c-Jun phosphorylation.

### NSL complex regulates c-Jun target gene expression

**Figure 3:**
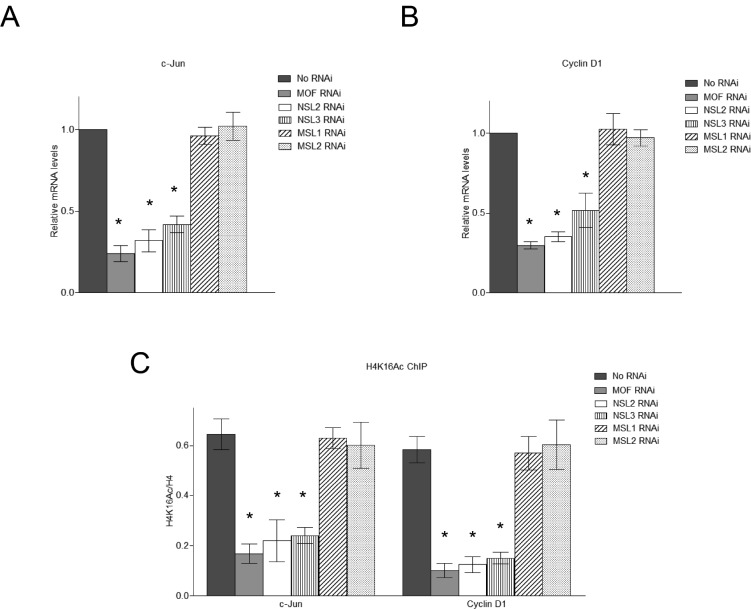
The NSL complex regulates the expression of c-Jun target genes **A.**, **B.** Depletion of the NSL complex subunits significantly reduces the expression of c-Jun target genes. HEK-293 cells were treated with siRNAs specific to MOF, NSL-specific subunits or MSL-specific subunits followed by treatment with Anisomycin. The expression levels of c-Jun and Cyclin D1 were measured by qPCR. The cells treated with only Anisomycin were used as a control. The data are presented as mean ± standard deviation of 3 independent experiments. *P < 0.01. **C.** Depletion of the NSL complex subunits significantly reduces the H4K16 acetylation levels at c-Jun target genes. HEK-293 cells were treated with siRNAs specific to MOF, NSL-specific subunits or MSL-specific subunits followed by treatment with Anisomycin. The resultant cells were then subjected to ChIP analysis using the indicated antibodies. The cells treated with only Anisomycin were used as a control. The data are presented as mean ± standard deviation of 3 independent experiments. *P < 0.01.

Having found that upon activation, c-Jun recruits the NSL complex to the promoters of its target genes, we next asked whether the NSL complex plays a role in c-Jun activated gene expression. To this end, we individually knocked down the expression of the subunits of the NSL complex in HEK-293 cells (Figure S4). Cells were then treated with Anisomycin for 30 minutes to activate c-Jun, and then subjected to ChIP analysis. The data showed that the knockdown of the subunits of NSL complex significantly impaired c-Jun target gene expression (Figure 3A and 3B), indicating a functional involvement of the NSL complex in c-Jun activated gene expression. In contrast, the knockdown of MSL complex-specific subunits did not affect c-Jun target gene expression (Figure 3A and 3B).

We further examined the H4K16 acetylation levels at the promoters of c-Jun target genes after depletion of the NSL complex. As shown in Figure 3C, the depletion of the NSL complex subunits significantly reduced the H4K16 acetylation levels at c-Jun target genes. We also examined the H4K16 acetylation levels at the promoters of c-Jun target genes upon the depletion of MSL complex specific subunits. The results showed that the knockdown of MSL complex specific subunits did not affect H4K16 acetylation levels at the promoters of c-Jun target genes (Figure 3C). These results further confirmed that the NSL complex, but not the MSL complex, is responsible for the H4K16 acetylation on the promoters of c-Jun target genes upon c-Jun activation.

### The NSL complex promotes the release of the repressive NuRD complex from c-Jun target gene promoter upon c-Jun activation

**Figure 4:**
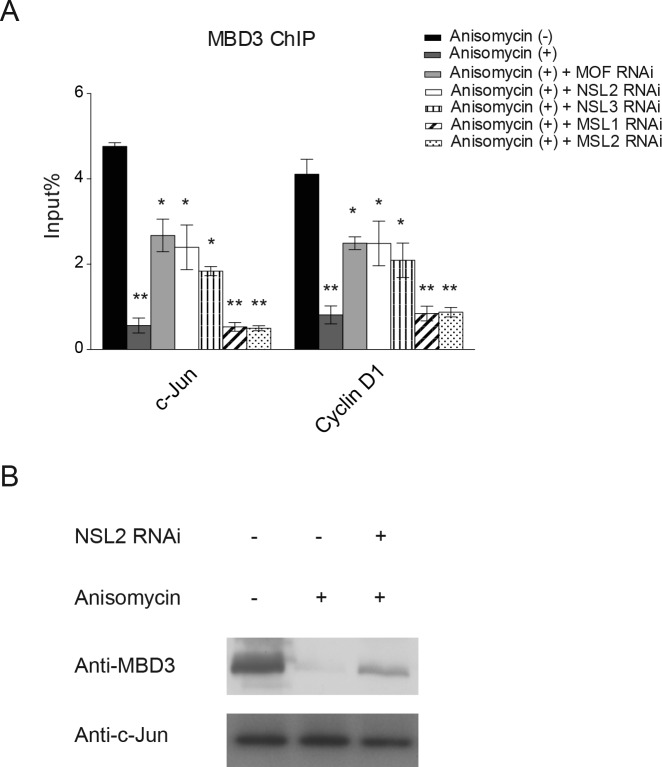
The NSL complex promotes the release of the NuRD complex from c-Jun target genes upon c-Jun activation **A.** HEK-293 cells were treated with siRNAs specific to MOF, NSL-specific subunits or MSL-specific subunits followed by treatment with Anisomycin. The resultant cells were then subjected to ChIP analysis using antibodies against MBD3, a subunit of the NuRD complex. The untreated cells or the cells treated with only Anisomycin were used as controls. The data are presented as mean ± standard deviation of 3 independent experiments. *P < 0.01, **P < 0.001. **B.** The depletion of NSL2 partially restores the interaction between c-Jun and MBD3. Wild-type HEK-293 cells or HEK-293 cells depleted of NSL2 were treated with Anisomycin as indicated. The resultant cells were then subjected to Co-IP analysis using antibodies against c-Jun.

The NuRD complex is a co-repressor for many transcription factors. It has been reported that c-Jun is physically associated with and repressed by the NuRD complex [35]. Upon activation, c-Jun is released from this repressive complex. In addition, the study found that c-Jun activation also prompts the dissociation of the NuRD complex from the promoters of c-Jun target genes [35]. Having found that c-Jun recruits the NSL complex to its target gene promoter upon activation, we then asked whether the NSL complex plays a role in the dissociation of the NuRD complex from the promoter of c-Jun target genes. To this end, we first individually knocked down the subunits of the NSL complex. The resultant cells were then treated with Anisomycin for 30 minutes to activate c-Jun before being subjected to a ChIP assay to measure the occupancy of MBD3, a subunit of NuRD complex, at the promoters of c-Jun target genes. As shown in Figure 4A, we observed a significant increase in MBD3 binding at the c-Jun target gene promoter upon the depletion of MOF. Similar results were observed when NSL complex specific subunits were depleted. However, the depletion of MSL-specific subunits did not alter the occupancy of MBD3 at the promoter of c-Jun target gene. These results demonstrate that upon activation, c-Jun recruits the NSL complex to promote the release of the NuRD repressor complex from the promoter of c-Jun target gene, thus activating c-Jun target gene transcription. This conclusion is strengthened by our biochemical data, which showed that the disruption of the NSL complex partially restored the interaction between c-Jun and MBD3 even in the presence of anisomycin (Figure 4B).

## DISCUSSION

Phosphorylation has been shown to be a critical post-translational modification in cellular signaling pathways, where it can act as a molecular switch to directly activate protein kinases or downstream transcription factors [38, 39]. In mitogen activated protein kinase (MAPK) signaling pathways, the downstream transcription factor c-Jun is activated by JNK-mediated phosphorylation in response to a variety of extracellular stimuli. Phosphorylation activates c-Jun by various mechanisms. First, c-Jun transcriptional activity is directly enhanced by its phosphorylation. In addition, c-Jun phosphorylation also promotes its release from the repressive NuRD complex. Here we identified the NSL complex, a MOF-containing complex, as a co-activator for c-Jun, and the recruitment of the NSL complex to the promoter of c-Jun target gene is dependent on c-Jun phosphorylation. Our findings reveal a new mechanism that regulates c-Jun activation, adding another layer to the mechanism of c-Jun activation regulation.

Two distinct MOF-containing complexes have been characterized in Drosophila and mammals, male-specific lethal (MSL) complex and non-specific lethal (NSL) complex [40, 41]. The two complexes show different genome binding patterns. In Drosophila, the MSL complex is primarily localized at the 3′-end of X-linked genes and catalyzes H4K16 acetylation to ensure equal expression of X-linked genes in both males and females. In contrast, the NSL complex has been shown to be enriched in the promoter region of more than 4000 target genes [38]. The depletion of the NSL complex subunits severely impairs its target gene expression in a genome-wide manner. The NSL complex has also been reported to globally associate with housekeeping genes and regulate a subset of them [41]. In addition, the NSL complex also regulates specific sets of expressed genes in mouse embryonic stem cell (mESC), as well as during differentiation [42]. Our results demonstrate that the NSL complex is involved in extracellular signal-induced c-Jun target gene expression. It is possible that the NSL complex also plays a regulatory role in the induction of additional genes.

Our data indicates that c-Jun recruits the NSL complex to the promoter of c-Jun target genes. At the promoter the NSL complex exerts its regulatory function in two ways: catalyzing H4K16 acetylation and promoting the release of the repressive NuRD complex. H4K16 acetylation has been shown to be enriched in both the promoter and the gene body of actively transcribed genes. This particular histone modification is important for gene transcription, as evidenced by many studies. For instance, in the dosage compensation phenomenon in Drosophila, the only X chromosome in males is highly enriched in H4K16 acetylation, and the expression from male X chromosomal genes is increased approximately by 2-fold. Our results suggest that the H4K16 acetylation catalyzed by the NSL complex at the promoter of c-Jun target genes may be essential for their activation. It should be pointed out that histone H4 may not be the only substrate for MOF. It is possible that MOF may directly acetylate c-Jun. It has been previously reported that the Drosophila c-Jun homologue, Jra, is acetylated by CBP and the acetylation regulates its stability [43]. Although the corresponding residue of the acetylated lysine of Drosophila Jra is glutamine in human c-Jun, it is possible that other lysine residues in human c-Jun are acetylated by MOF and the acetylation might directly regulate c-Jun transcriptional activity or regulate its interaction with other factors. Similarly, the MOF in the NSL complex might also acetylate specific subunits of the NuRD complex, which may lead to its release from the promoter of c-Jun target genes. Future investigations will be needed to address these possibilities.

**Figure 5:**
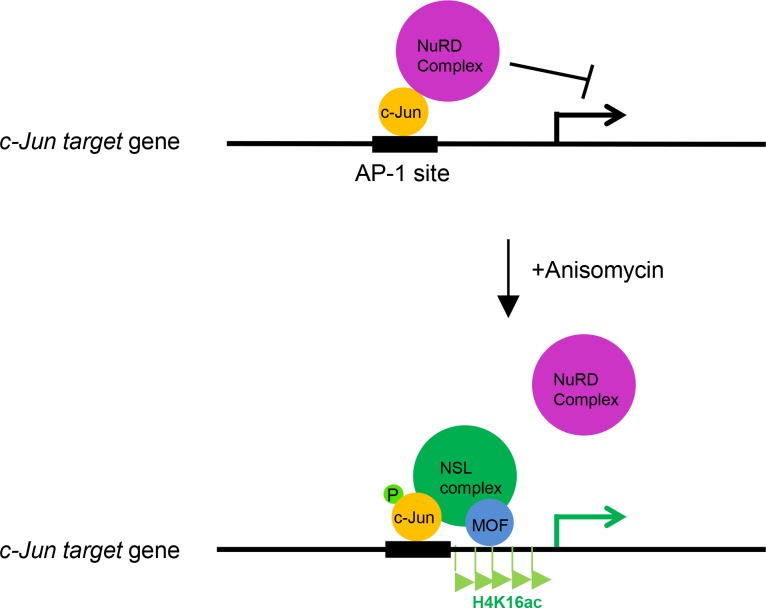
The working model depicting the role of the NSL complex in c-Jun activated gene expression c-Jun recruits the NSL complex to its target genes upon activation. The NSL complex catalyzes H4K16 acetylation to promote c-Jun target gene transcription and promotes the release of the repressive NuRD complex from the c-Jun target genes, thus activating c-Jun.

In summary, our results reveal an important regulation mechanism for c-Jun activation. Based on our data, we proposed a model, in which c-Jun recruits the MOF-containing NSL complex to its target gene promoters in response to extracellular stimuli, and the NSL complex promotes the activation of c-Jun target genes in two ways: catalyzing H4K16 acetylation and promoting the releases of the repressive NuRD complex (Figure 5).

## MATERIALS AND METHODS

### Cell culture and transfection

Human embryonic kidney cell line HEK-293 cells were maintained in Dulbecco's modified Eagle medium (DMEM) supplemented with 10% fetal bovine serum (FBS) and 1% penicillin-streptomycin (PS) at 37°C in a humid atmosphere of 5% CO_2_. For RNA interference (RNAi) knockdown experiments, siRNAs were transfected into HEK-293 cells with Lipofectamine 2000 reagent (Invitrogen) following the manufacturer's manual. After 48 hours, cells were harvested and subjected to Western blot analysis to examine the knockdown efficiency. To inhibit endogenous JNK, HEK-293 cells were treated with 50 μM JNKi (SP600125, Calbiochem). HEK-293 cells were treated with 25 ng/ml Anisomycin (Sigma) for 30 minutes to activate c-Jun.

### Immunoprecipitation

HEK-293 cells were pooled and washed with ice-cold phosphate buffered saline (PBS), and then lysed for 30 min at 4°C in lysis buffer containing 100 mM Tris-Cl [pH 7.5], 150 mM NaCl, 1 mM EDTA, 1% NP-40, 100 μM Na-orthovanadate, 0.25 mM phenylmethylsulfonyl fluoride (PMSF), and protease inhibitor cocktail (Sigma) in PBS. Cell lysates were incubated overnight at 4°C with 1 μg of the anti-c-Jun (CST, 2315) antibody. After incubation with antibody, Protein A/G-agarose beads (Santa Cruz, sc-2003) were added into the reaction and incubated for another four hours. The resultant agarose beads were washed five times with lysis buffer. The precipitated proteins were then analyzed by Western blot assay.

### Chromatin immunoprecipitation (ChIP)

ChIP analysis was performed according to a previously published protocol [44]. The resultant ChIP DNA samples were analyzed by quantitative real-time PCR (qRT-PCR) using the following primers: Cyclin D1 ChIP F, 5′-CCA ATT AGG AAC CTT CGG TGG TC-3′; Cyclin D1 ChIP R, 5′-GGT GGC CAG CAT TTC CTT CAT C-3′; c-Jun ChIP F, 5′-AAG AAT CTT CTA GGG TGG AG-3′; c-Jun ChIP R, 5′-GAG CCC TTA TCC AGC CCG AG-3′. qRT-PCR was performed with RealMasterMix (SYBR Green; Tiangen FP202) using a BioRad iQ5 (BioRad).

### RNA extraction and qRT-PCR analysis

Total mRNA was isolated using Trizol reagent according to the manufacturer's instructions (Invitrogen). Complementary DNA (cDNA) was synthesized by reverse transcription using Invitrogen Superscript reagents according to the manufacturer's instructions. Primers used in qRT-PCR are as follows: c-Jun F, 5′-TCG ACA TGG AGT CCC AGG A-3′; c-Jun R, 5′-GGC GAT TCT CTC CAG CTT CC-3′; Cyclin D1 F, 5′-GCG CGT ACC CCG ATG CCA AC-3′; Cyclin D1 R, 5′-CTC GCA GAC CTC CAG CAT CCA-3′; β-actin F, 5′-GGA TGC AGA AGG AGA TCA CTG-3′; β-actin R, 5′-CGA TCC ACA CGG AGT ACT TG-3′. qRT-PCR was performed with RealMasterMix (SYBR Green; Tiangen FP202) using a BioRad iQ5 (BioRad). Housekeeping gene *β-actin* was used as an internal control for normalization.

## SUPPLEMENTARY MATERIAL FIGURES


