# Supplementary material for: C-Jun recruits the NSL complex to regulate its target gene expression by modulating H4K16 acetylation and promoting the release of the repressive NuRD complex
Source: Oncotarget. 2015 May 4;6(16):14497–506. doi: 10.18632/oncotarget.3988 (PMC4546482; doi:10.18632/oncotarget.3988)
Supplement: Supplementary file 1 [file oncotarget-06-14497-s001.pdf]

C-Jun recruits the NSL complex to regulate its target gene expression by modulating H4K16 acetylation and promoting the release of the repressive NuRD complex

Supplementary Material

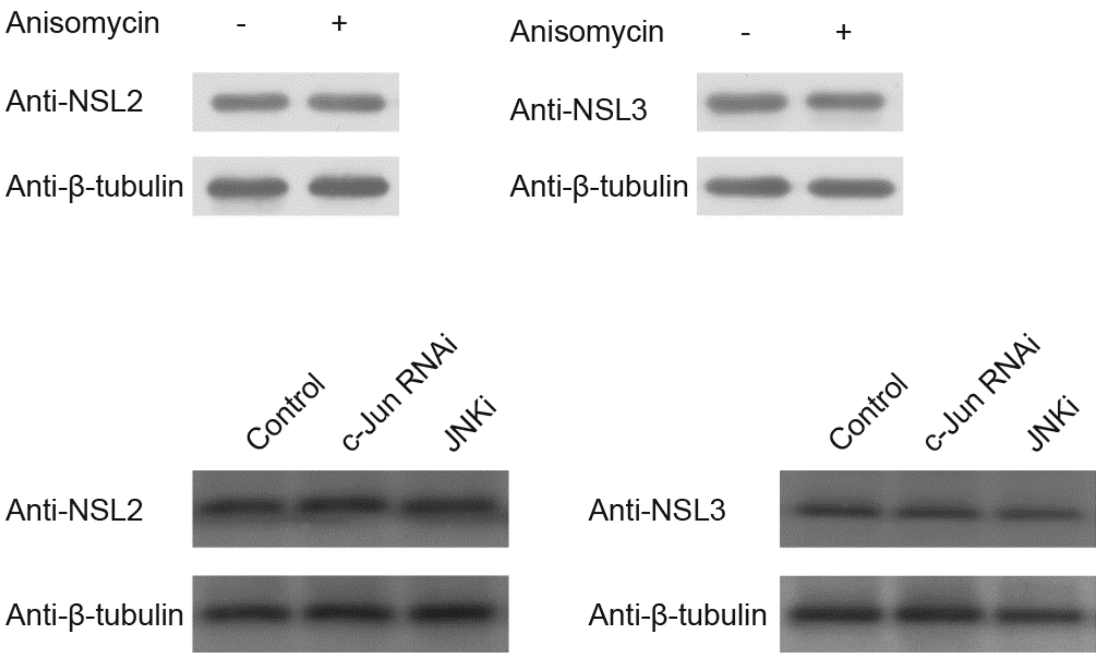

Figure S1: Western blot assay was performed to confirm that there is no significant change in protein levels of the NSL complex components under the conditions used in this study.

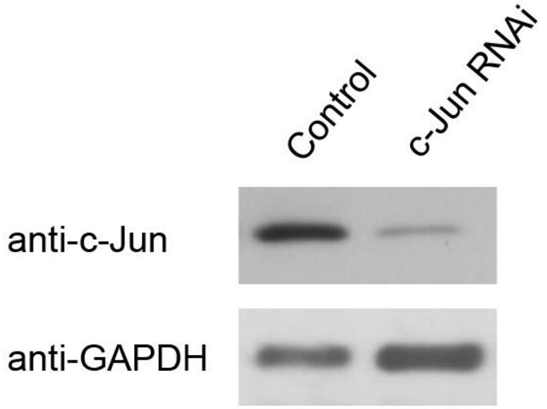

Figure S2: Western blot assay was performed to verify c-Jun knockdown efficiency.

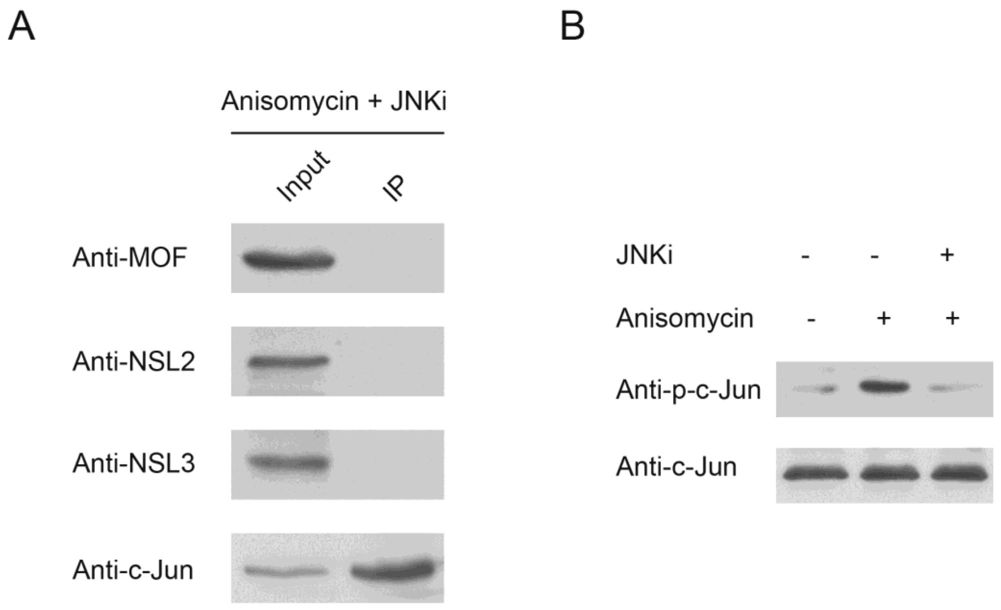

Figure S3: C-Jun phosphorylation is required for the interaction of c-Jun with the NSL complex. **A.** C-Jun does not co-precipitate with the NSL complex after JNKi treatment. **B.** Anisomycin activates c-Jun and the JNKi treatment greatly reduces c-Jun phosphorylation.

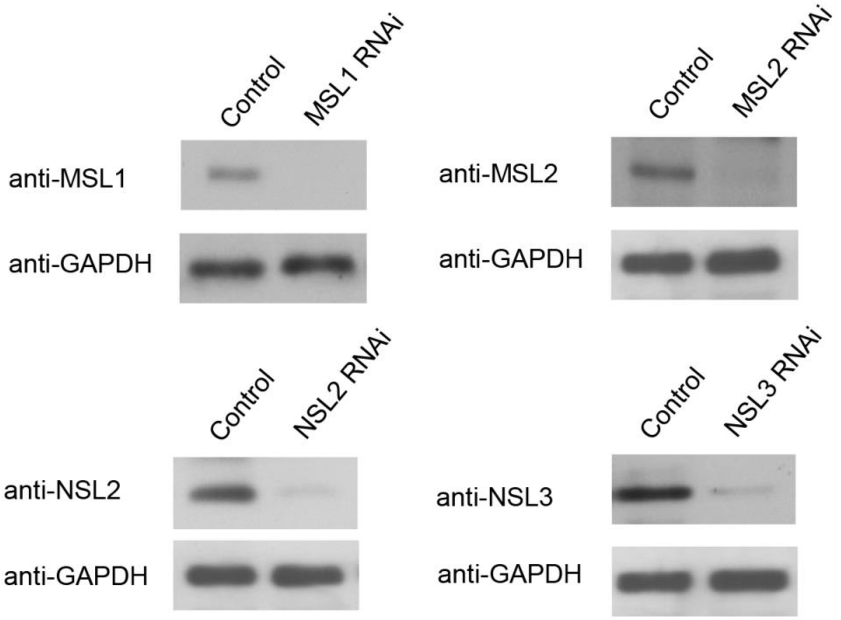

Figure S4: Western blot assay was performed to verify knockdown efficiency of NSL2, NSL3, MSL1 and MSL2.
